# First report and molecular identification of *Opisthorchis viverrini* infection in human communities from Lower Myanmar

**DOI:** 10.1371/journal.pone.0177130

**Published:** 2017-05-04

**Authors:** Win Pa Pa Aung, Thi Thi Htoon, Htay Htay Tin, Kyi Kyi Thinn, Oranuch Sanpool, Jurairat Jongthawin, Lakkhana Sadaow, Issarapong Phosuk, Rutchanee Rodpai, Pewpan M. Intapan, Wanchai Maleewong

**Affiliations:** 1 Department of Parasitology, Faculty of Medicine and Research and Diagnostic Center for Emerging Infectious Diseases, Mekong Health Science Research Institute, Khon Kaen University, Khon Kaen, Thailand; 2 Department of Microbiology, University of Medicine 2, Ministry of Health and Sport, Yangon, Myanmar; 3 Department of Parasitology, National Health Laboratory, Yangon, Myanmar; 4 International Relation Division, University of Medicine 1, Ministry of Health and Sport, Yangon, Myanmar; 5 Faculty of Medicine, Mahasarakham University, Mahasarakham, Thailand; Charles University, CZECH REPUBLIC

## Abstract

*Opisthorchis viverrini* is endemic in the South East Asian region, especially in Cambodia, Lao People's Democratic Republic, Vietnam and Thailand, but there have been no previous records from Myanmar. During stool surveys of rural populations in three regions of Lower Myanmar, *Opisthorchi*s-like eggs were found in 34 out of 364 (9.3%) participants by stool microscopy after using the modified formalin-ether concentration technique. DNA was extracted from these positive stool samples and a portion of the mitochondrial cytochrome c oxidase subunit I (*cox1*) gene was amplified using the polymerase chain reaction and then sequenced. DNA sequences, successfully obtained from 18 of 34 positive samples (Bago Region, n = 13; Mon State, n = 3; Yangon Region, n = 2), confirmed that the eggs were of *O*. *viverrini*. Sequences showed 99.7% identity with *O*. *viverrini* mitochondrial *cox1* (GenBank accession no. JF739555) but 95%, 88.7%, 82.6% and 81.4% identities with those of *Opisthorchis lobatus* from Lao People's Democratic Republic (GenBank accession nos. HQ328539-HQ328541), *Metorchis orientalis* from China (KT239342), *Clonorchis sinensis* from China (JF729303) and *Opisthorchis felineus* from Russia (EU921260), respectively. When alignement with other Opisthorchiidae trematodes, 81% similarity with *Metorchis bilis* from Czech Republic (GenBank accession nos. KT740966, KT740969, KT740970) and Slovakia (GenBank accession nos. KT740971-KT740973), 84.6% similarity with *Metorchis xanthosomus* from Czech Republic (GenBank accession no. KT740974), 78.6% similarity with *M*. *xanthosomus* from Poland (GenBank accession no. KT740968) and 82.2% similarity with *Euamphimerus pancreaticus* from Czech Republic (GenBank accession no. KT740975) were revealed. This study demonstrated, for the first time, *O*. *viverrini* from rural people in Myanmar using molecular methods and is an urgent call for surveillance and control activities against opisthorchiasis in Myanmar.

## Introduction

*Opisthorchis viverrini*, a fish-borne liver fluke, is one of the main public health problems in the South East Asian region, especially along the Mekong River (Cambodia, Lao People's Democratic Republic, Vietnam, and Thailand), where there is a culture of eating raw or undercooked fish that contain encysted metacercariae of the liver fluke [1–3]. Adult flukes are long-lived and feed on epithelial cells of the intrahepatic bile ducts. Although most cases are asymptomatic, complications can include hepatobiliary diseases such as hepatomegaly, cholangitis, cholecystitis, peri-ductal fibrosis and gallstones. Severe chronic infection is a strong risk factor for cholangiocarcinoma [4–6]. More than ten million people are estimated to be infected with *O*. *viverrini*: about eight million in Thailand and two million in Lao People's Democratic Republic [7]. The highest prevalences occur in North and Northeast Thailand, especially in rural populations [2,8–9] and in the adjacent southern and central regions of Lao People's Democratic Republic [10–11]. Human infections with *O*. *viverrini* and presence of metacercariae in intermediate hosts have been reported in several provinces of Cambodia [12–15]. Miyamoto et al. [13] reported *O*. *viverrini* eggs in human fecal samples from 26 out of 55 surveyed villages in five provinces of Cambodia, among which 15 villages had an egg positive rate >10%. Polymerase chain reaction (PCR) amplification and DNA sequencing confirmed the presence of *O*. *viverrini* in 30 samples from 5 villages. The parasite is also endemic in southern and central parts of Vietnam [16–17]. A survey conducted in 2015 reported that the overall prevalence of *O*. *viverrini* infection was 11.4% in central Vietnam [16]. Although not reported to date from Myanmar, the presence of *O*. *viverrini* is likely because of its close proximity to endemic areas in Thailand and because of an open-borders policy that started in 2015 leading to increasing migration among ASEAN Economic Community (AEC) countries (Thailand, Lao People's Democratic Republic, Cambodia, Vietnam and Myanmar) [18].

Opisthorchiasis is usually diagnosed by microscopic examination of eggs in feces, bile or duodenal fluid. However, it is difficult to differentiate opisthorchiid eggs morphologically from those of other trematodes such as lecithodendriids and heterophyids, prompting the development of molecular approaches for this purpose [19–20]. A lack of awareness of potential *O*. *viverrini* infection in Myanmar will hinder prevention and control programs against this fluke. This study aims to identify and confirm the presence of *O*. *viverrini* in rural parts of Myanmar using conventional and molecular methods. Thus, the results of this study will be helpful to health authorities and researchers in Myanmar for diagnosis of opisthorchiasis and for planning and implementing control strategies.

## Materials and methods

### Study design and population

**Fig 1:**
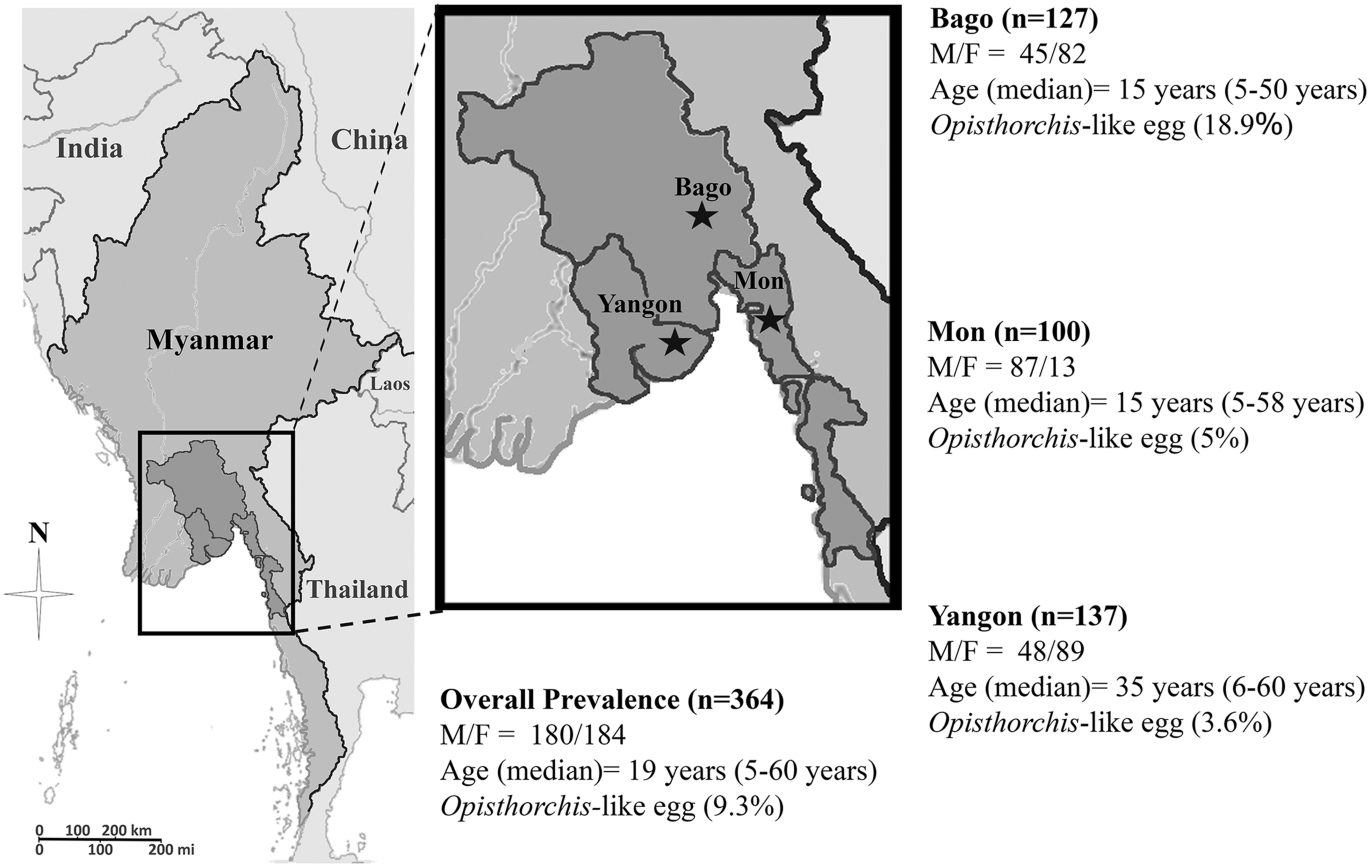
Map of the survey areas in Myanmar. Prevalences (in parentheses) of *Opisthorchis*-like eggs in Bago, Mon and Yangon Regions in the years 2015–2016. M = Male; F = Female. The Myanmar map was modified from a map in The World Factbook, published by the Central Intelligence Agency [22].

Stool surveys for intestinal parasites in rural populations in three regions of Lower Myanmar (Fig 1) were conducted between June 2015 and March 2016. Sample-size calculation was performed using simple random sampling based on preliminary prevalence data for soil-transmitted helminthiasis [21]. This study was approved by the Research and Ethical Committee of the University of Medicine 1, Yangon, Myanmar (5695/ Research and Ethics 2015) and Khon Kaen University Ethics Committee for Human Research (HE581396). Stool samples were collected from 5 years old up rural people (select the age group has occasion to contact with the soil) including school children, housewife, farmers, plantation workers and gold miners; and excluded the persons who received anthelmintic treatment within 6 months prior to study and persons presenting with diarrhoea at the time of sampling.

Each participant was informed of study procedures, risks and benefits of the process. Before enrolment, written consent was obtained from all adult participants and from parents or legal guardians of minors.

### Stool examination and parasite collection

**Fig 2:**
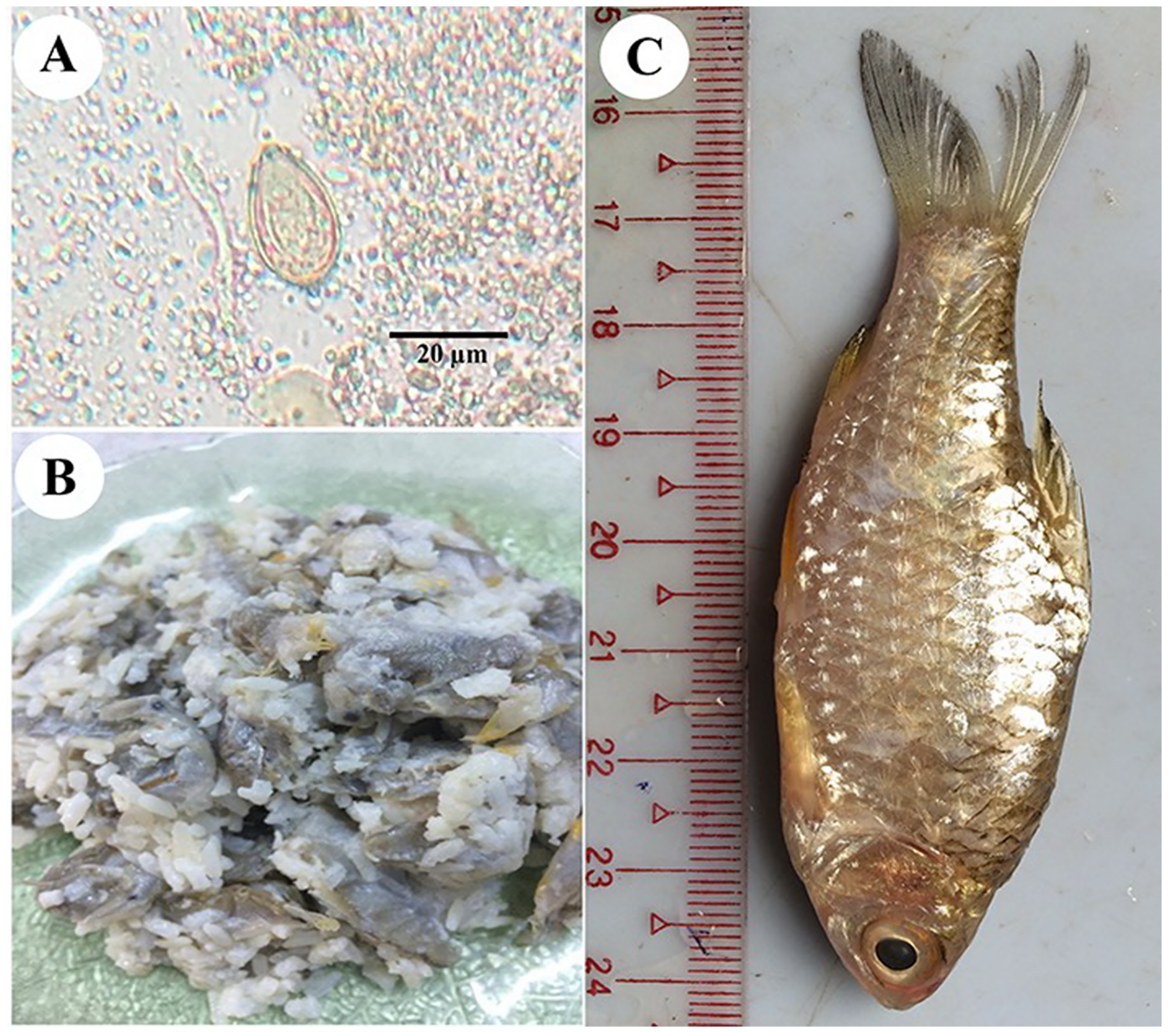
Showing *O*. *viverrini* egg, fish dish and cyprinoid fish. A) *Opisthorchis viverrini* egg from stool microscopy. B) The undercooked or fermented fish dish (small raw fish fermented with cooked rice and salt) named “Ngar Lay Chin”. C) Cyprinoid fish (*Henicorhynchus* sp.) “Nga Khone Ma” (intermediate host of *O*. *viverrini*).

Stool examination was done using the modified formalin-ether concentration technique at National Health Laboratory, Yangon, Myanmar [23]. Geometric Mean fecal egg counts, expressed as eggs per gram (epg) of feces, were used to indicate intensity of helminth infection. Given the difficulty of distinguishing eggs of many trematode species, such eggs were initially recorded as being *Opisthorchis*-like (Fig 2A). Samples containing these eggs were preserved in 70% alcohol in a 15 ml centrifuge tubes and shipped to Khon Kaen University for molecular identification.

### DNA extraction, amplification and sequencing

DNA was extracted from the stool samples positive for *Opisthorchis*-like eggs using a QIAamp^®^ DNA stool mini kit (Qiagen, Hilden, Germany), according to the manufacturer’s instructions. The DNA was eluted in 50 μl of elution buffer, 5 μl of which was used for the PCR reaction. New PCR primers were designed to bind to a conserved portion of the mitochondrial *cox1* gene of *O*. *viverrini* (GenBank accession number JF739555): COIP4bF (5´- GGGACCCGATTATGTGGTGG- 3´) as a forward primer and OvCOIP4bR (5´- CCCACTGAAAACAAGGCTCG-3´) as a reverse primer, producing an amplicon of about 348 base pairs.

The PCR products were amplified in a GeneAmp PCR system 9700 thermal cycler (Applied Biosystems, Singapore) using the following PCR conditions: initial denaturation at 94°C for 5 min followed by 35 cycles of denaturation at 95°C for 30 seconds, annealing at 62°C for 30 seconds and 72°C for 30 seconds, followed by a post-amplification extension for 10 min at 72°C. The PCR product was visualized in a 1% agarose gel to confirm and estimate amplified product length and quantity. DNA sequencing was done in both directions, using the PCR primers as sequencing primers, with an Applied Biosystems 3730 +I DNA Analyzer and an ABI Big Dye Version 3.1 cycle sequencing kit (Foster City, CA). The nucleotide sequences were compared with sequence data for *O*. *viverrini*, *Clonorchis sinensis*, *Opisthorchis felineus*, and *Metorchis orientalis* in GenBank by BLAST-N search via NCBI. DNA sequences were aligned using ClustalW. All sequences have been submitted to the GenBank database (GenBank accession nos. KX922693-KX922699).

### Statistical analysis

Data were analyzed using SPSS version 22 (SPSS Inc., Chicago, IL) and expressed as numbers and as percentages. Chi-square test was applied to investigate the correlation between parasite infection status and sex. A *p*-value of less than 0.05 was regarded as statistically significant. For each population, the geometric means and ranges of eggs per gram of faeces were used to express the intensity of infection.

## Results

### Prevalence of *Opisthorchis*-like eggs in the study regions by microscopic examination of stool samples

A total of 364 study participants submitted their stool sample for analysis. Of these, 180 (49.5%) were males with median age of 19 years (ranged 5 to 60 years). One-hundred participants were from Mon state, 127 from Bago region and 137 from Yangon (Fig 1). The overall prevalence of *Opisthorchis*-like eggs was 9.3% with the highest positive rate in the Bago Region (18.9%), followed by Mon State (5%) and Yangon Region (3.6%) (Fig 1). Infection rates did not differ significantly between males and females in any study area. However, a trend towards a higher rate in females (odds ratio 0.53, 95% CI 0.25–1.1). Prevalences differed according to age, with a non-significant trend towards higher prevalences in older people in each study area (Table 1). Fecal counts for *Opisthorchis*-like eggs ranged from 10 to 150 epg (Geometric Mean = 22.63 epg).

**Table 1:** Prevalence and intensity of *Opisthorchis*-like eggs by sex and age group in the three study areas.

	Address			
	Bago n (%)	Mon n (%)	Yangon n (%)	Overall n (%)
**Egg positive cases**	24/127 (18.9)	5/100 (5)	5/137 (3.6)	34/364 (9.3)
**Sex Male**	7/45 (15.6)	3/87 (3.4)	2/48 (4.2)	12/180 (6.7)
** Female**	17/82 (20.7)	2/13 (15.4)	3/89 (3.4)	22/184 (12)
*p*-value*	0.476	0.125	1.000	0.083
**Age group**				
**5–11 Years**	5/42 (11.9)	0/15 (0)	0/9 (0)	5/66 (7.5)
**12–19 Years**	5/27 (18.5)	2/70 (2.9)	1/21 (4.8)	8/118 (6.8)
**20–40 Years**	7/34 (20.6)	1/5 (20.0)	3/59 (5.1)	11/98 (11.2)
**>41 Years**	7/24 (29.2)	2/10 (20.0)	1/48 (2.1)	10/82 (12.2)
**Intensity** (Geometric mean, range of eggs per gram)	19.91, 10–90	32.72, 10–150	28.95, 10–44	22.63, 10–150

* *p- value* less than 0.05 was considered statistically significant

n = number of positive cases/number of total participants

### Molecular identification of *O*. *viverrini*

**Fig 3:**
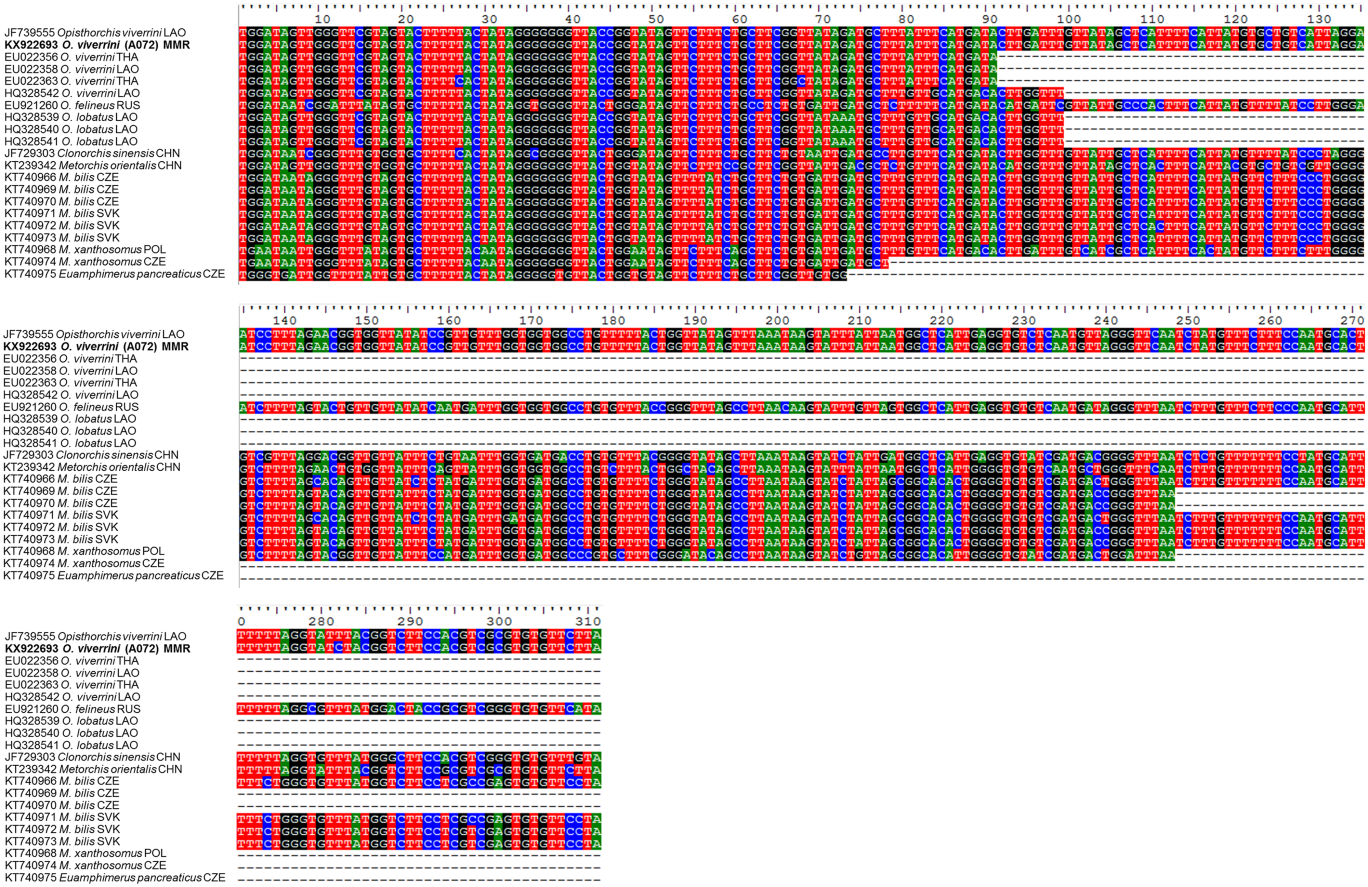
Alignment of *O*. *viverrini cox1* nucleotide sequences. The multiple sequences alignment of representative *O*. *viverrini* partial mitochondrial cytochrome c oxidase subunit I (*cox1*) sequence from Bago Region of Lower Myanmar (KX922693; bold letters) and other Opisthorchiidae trematodes. A, T, G, C nucleotides represent in green, red, black and blue, respectively. Incomplete sequences are represented by dashed lines (-). ISO 3166 Countries Codes are presented (THA, Thailand; LAO, Lao People's Democratic Republic, MMR; Myanmar, VNM; Vietnam, RUS; Russian Federation, CHN; China, CZE; Czech Republic, SVK; Slovakia and POL; Poland).

DNA from stool samples that were positive for *Opisthorchis*-like eggs (n = 34) was used for molecular identification using primers specific for the mitochondrial *cox1* gene. DNA sequences, successfully obtained from 18 of 34 positive samples (Bago Region, n = 13; Mon State, n = 3; Yangon Region, n = 2), confirmed that the eggs were of *O*. *viverrini*. All partial mitochondrial *cox1* gene sequences obtained shared 99.7% similarity with that of *O*. *viverrini* (complete mitochondrial genome—GenBank accession no. JF739555). There were also high similarities with *O*. *viverrini cox1* sequences from Lao People's Democratic Republic (96% similarity, coverage 31.8%, GenBank accession no. HQ328542 and 100% similarity, coverage 29.3%, GenBank accession no. EU022358) and Thailand (100% similarity, coverage 29.3%, GenBank accession no. EU022356 and 97.8% similarity, coverage 29.3%, GenBank accession no. EU022363) (Fig 3) Comparisons were also made with other species of Opisthorchiidae trematodes. Our sequences from Myanmar had only 95% similarity with *Opisthorchis lobatus* from Lao People's Democratic Republic (GenBank accession nos. HQ328539-HQ328541), 82.6% similarity with *C*. *sinensis* from China (GenBank accession no. JF729303), 81.4% similarity with *O*. *felineus* from Russia (GenBank accession no. EU921260), and 88.7% similarity with *M*. *orientalis* from China (GenBank accession no. KT239342), 81% similarity with *Metorchis bilis* from Czech Republic (GenBank accession nos. KT740966, KT740969, KT740970) and Slovakia (GenBank accession nos. KT740971-KT740973), 84.6% similarity with *Metorchis xanthosomus* from Czech Republic (GenBank accession no. KT740974), 78.6% similarity with *M*. *xanthosomus* from Poland (GenBank accession no. KT740968) and 82.2% similarity with *Euamphimerus pancreaticus* from Czech Republic (GenBank accession no. KT740975).

## Discussion

Since the eggs of *O*. *viverrini* are difficult to discriminate morphologically from those of small intestinal flukes, PCR-based amplification and sequencing of a partial fragment of the mitochondrial *cox1* gene have been used for differentiation of *Opisthorchis*-like eggs in previous studies [24–25]. The present study demonstrated for the first time the presence of *O*. *viverrini* from fecal samples of rural people in Myanmar by this molecular method. PCR products were only obtained from 18 of 34 fecal samples in which *Opisthorchis*-like eggs were found. There are several explanations for this. The number of eggs (and hence DNA yield) might have been too low in some samples for amplification to occur; the eggs observed might have been shed by members of other trematode families such as Heterophyidae and Lecithodendriidae; inhibitors in some fecal samples might have blocked amplification.

Opisthorchiasis is endemic in neighboring countries throughout the Mekong Basin, including Thailand, Lao People's Democratic Republic, Cambodia and Vietnam [9] but was not previously known from Myanmar. Using molecular methods, we confirmed opisthorchiasis in 18 individuals (4.9%) in rural populations in our study areas. This is considered a low prevalence [9], but our survey was limited and must be regarded as preliminary. In agreement with studies done in Lao People's Democratic Republic and Cambodia, our study found that prevalence did not significantly differ between the sexes but did tend to increase with age [13, 26]. *Opisthorchis* infection is highly localized, depending on the food habits of local people and the presence of susceptible snail hosts [2]. Prevalence of *O*. *viverrini* was much higher in the Bago Region than elsewhere, possibly owing to the local habit of eating raw or semi-cooked or fermented fish dishes (named “Ngar Lay Chin”) in that region (Fig 2B and 2C). The eating of raw or undercooked fish is a known risk factor for opisthorchiasis [26]. Infection can also occur via the contamination of food, hands, surfaces and food preparation utensils with the infective stage of the parasite (i.e., metacercariae) [26]. A high prevalence of *O*. *viverrini* infection is strongly positively correlated with the incidence of cholangiocarcinoma in Lao People's Democratic Republic and endemic areas of Thailand [26]. Although human opisthorchiasis control programs have been conducted in endemic areas in the Mekong Basin for a long time, the infection still prominent in many areas [9]. Although opisthorchiasis caused by *O*. *viverrini* is common in South East Asian region especially in greater Mekong sub-region countries, we should be aware of possibility of minor agents of Opisthorchiidae trematodes such as *O*. *lobatus*, *O*. *felineus*, *Opisthorchis noverca*, *M*. *orientalis*, *M*. *bilis*, *M*. *xanthosomus*, and *E*. *pancreaticus* since they are closely related and cannot be easily differentiated with molecular method [27–30]. Understanding of the biology, distribution, transmission and factors influencing *O*. *viverrini* infection is required to help establish sustainable human liver fluke prevention and control programs.

## Conclusion

In conclusion, we have reported for the first time the presence of *O*. *viverrini* in rural people from Myanmar. Identity of the parasite was confirmed by molecular methods. Since there is no accurate diagnosis and reporting of *O*. *viverrini* infections in Myanmar, hospital-based studies and larger-scale nation-wide epidemiological studies should be conducted to assess the public health impact. In addition, the roles of intermediate hosts (snails and fish) and definitive hosts (dogs and cats) in transmission of this food-borne trematode infection in Myanmar should be investigated. Combating this disease of poor sanitation should be the focus of a jointly arranged action from government and community sectors.
